# Hematogenous metastasis and tumor dormancy as concepts or dogma? The continuum of vessel co-option and angiotropic extravascular migratory metastasis as an alternative

**DOI:** 10.3389/fonc.2022.996411

**Published:** 2022-10-11

**Authors:** Claire Lugassy, Hynda K. Kleinman, Nathalie Cassoux, Raymond Barnhill

**Affiliations:** ^1^Department of Translational Research, Institut Curie, Paris, France; ^2^Laboratory of Cell Biology, National Institute of Dental and Craniofacial Research (NIDCR), National Institutes of Health (NIH), Bethesda, MD, United States; ^3^University of Paris Réné Descartes Faculty (UFR) of Medicine, Paris, France; ^4^Department of Ophthalmology, Institut Curie, Paris, France

**Keywords:** tumor dormancy, hematogenous metastasis, extravascular migratory metastasis, angiotropism, drug resistance, dogma, gaps in knowledge

## Abstract

It has been accepted for many years that tumor cells spread *via* the circulation to distant sites. The latency period between treatment and tumor recurrence has been attributed to dormant cells in distant organs that emerge and grow as metastatic tumors. These processes are accepted with an incomplete demonstration of their existence. Challenging such a well-established accepted paradigm is not easy as history as shown. An alternative or co-existing mechanism involving tumor cell migration along the outside of the vessels and co-option of the blood vessel has been studied for over 25 years and is presented. Several lines of data support this new mechanism of tumor spread and metastatic growth and is termed angiotropic extravascular migratory metastasis or EVMM. This slow migration along the outside of the vessel wall may explain the latency period between treatment and metastatic tumor growth. The reader is asked to be open to this possible new concept in how tumors spread and grow and the reason for this latency period. A full understanding of how tumors spread and grow is fundamental for the targeting of new therapeutics.

## Introduction

**Figure 1:**
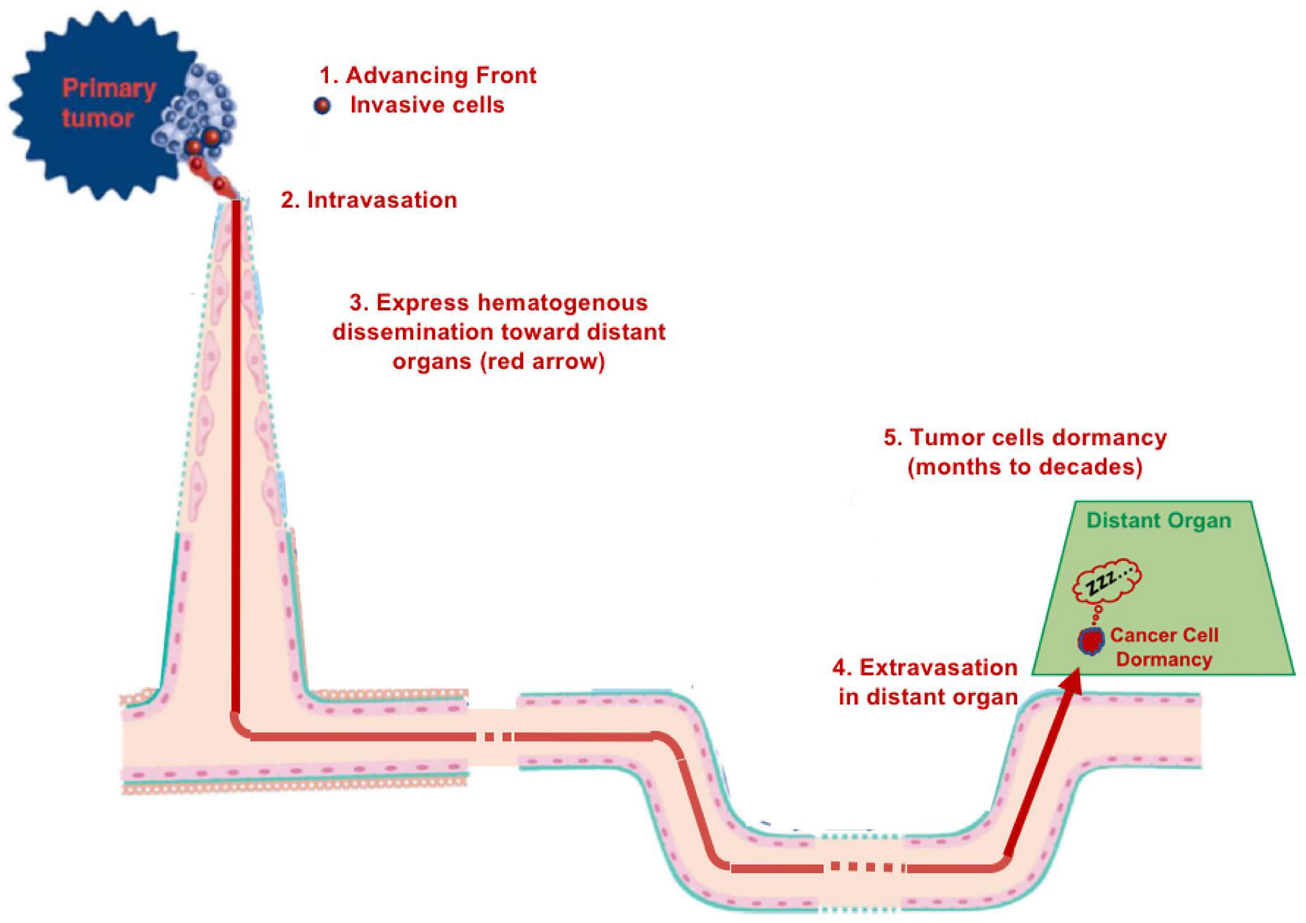
Diagram of hematogenous metastasis.

Metastasis is based on the main concept of hematogenous tumor spread (1) (**Figure 1**). This almost universally recognized metastatic cascade has been developed, modified, and enriched over time *via* new concepts or paradigms, in order to justify intravascular cancer dissemination, and to fill “gaps in our knowledge”. Metastasis was and is still considered inefficient (2) with many cells being ‘‘seeded’ through the circulation but only a few surviving because of mechanical damage due to shear stress (3), to anoikis or immune destruction (4). Moreover, as highlighted by IJ Fidler in 2010: “Despite almost 200 years of study, the process of tumor metastasis remains controversial” (5). Furthermore, even less is known about the proposed phenomenon of dormancy. Currently, dormancy is an hypothesis to explain the latency between tumor cell hematogenous dissemination from primary to secondary sites and the formation of metastases at these sites, and the perfect relevant model of dormancy has not yet been developed (6, 7). Some studies have identified putative dormant tumor cells in tissues and even a cell line is reported to have been isolated (8). If dormancy exists, it may be operative for only certain tumor types and not constitute the main mechanism of tumor spread for other tumor types (8). In addition, such “dormant cells” may correspond to cancer cells migrating slowly toward or within metastatic sites (9).

With the emergence of molecular biology in the 1950’s through the discovery of the structure of DNA, some researchers thought that the biological sciences could be reduced to the field of chemistry (10). The claim made by Francis Crick (1966) that “the ultimate aim of the modern movement in biology is to explain all biology in terms of physics and chemistry” is the perfect example of reductionism in molecular biology for over 70 years (11). Genetic reductionism maintains that all of biology is based on genes because the genome provides all of the codes of biological processes. According to this claim, genes are the foundation of the organism. In this type of reductionism, mathematical notions of information, program, and signal are critical (10). By the beginning of the 21^st^ century, with the increasing significance of developmental biology, some researchers questioned the reductionism in molecular biology (12). In contrast, as shown by an ever-increasing number of publications, the genomic perspective virtually controls the field of biomedical research (13). With the first publication of the sequencing of the human genome, many researchers aggressively embraced the reductionist agenda that gave rise to precision medicine (Genome International Sequencing Consortium 2001) (12, 14).

Efforts at proving the reality of the “inefficient hematogenous metastasis” (5), as well as “cancer dormancy”, remain challenging, because experimental studies and conceptual models cannot yet be validated in patients (7).

Thus, the present work has the following goals:

- To present brief historical and philosophical perspectives on hypothesis, paradigm, and dogma, in order to suggest that an accepted concept could be revisited. This will be illustrated with the disproved concept/dogma of Spontaneous Generation during the Pasteurian revolution.
- To examine intravascular cancer dissemination and cancer dormancy in a historical and scientific context, and show that they are accepted but still not proven concepts despite the accumulation over time of molecular data, *in vitro* systems, and animal models.
- To briefly describe a potential alternative to intravascular cancer dissemination and cancer dormancy with the now accepted alternative pathway of extravascular migratory metastasis (EVMM), in particular angiotropic EVMM.

### Hypothesis, paradigm, and dogma

As human beings, we have a tendency (i) to be misled by prejudices; (ii) to accept with time hypotheses as realities if not dogma; and (iii) to be anthropocentric in all domains, including sciences. These tendencies were already well described by great thinkers and philosophers such as Baruch Spinoza (15), Frederic Nietzsche (16, 17), and Thomas Kuhn (18, 19),

Probably Spinoza was the first to emphasize the role of superstition in dogmatism, by denouncing the finalist prejudice in his most famous book *Ethics*, written between 1661 and 1675 and first published posthumously in 1677 (15). “We must not omit to notice that the followers of this doctrine, anxious to display their talent in assigning final causes, have imported a new method of argument in proof of their theory –namely, a reduction, not to the impossible, but to ignorance; thus, showing that they have no other method of exhibiting their doctrine.” Again, in the appendix of the first part of his *Ethics*, Spinoza denounces an anthropomorphic interpretation of nature, that is, a reading of natural phenomena through the prism of human action that pursues ends. Interestingly, concerning modern cancer biology, we are still using an anthropomorphic vocabulary to describe “the will” of the cancer cell. For example, malignant cell invasion, destruction, etc.

Similarly, in *Beyond Good and Evil*, Nietzsche suggests that the foundation of all dogmatism is based on naive superstitions and prejudices. He cites as examples the “soul superstition” which remains even in atheistic philosophy as the “subject and ego superstition” (16). “Often, our *truths* are born from our prejudices, our will to deceive, and our falsehoods. We establish a number of old prejudices called *truths* and a whole system of philosophy is constructed after the fact to justify these *truths*”. In Book Five, “We Fearless Ones” (17), Nietzsche challenges such ideas and asks: “what happens when we de-deify nature and naturalize humanity?” (17). Paraphrasing Nietzsche, we question: “what happens when we reduce biology to chemistry and denaturalize humanity?”

More recently in 1982, Thomas Kuhn challenged the then prevailing view of progress in science in which scientific progress was viewed as “development-by-accumulation” of accepted facts and theories (18, 20). Kuhn proposed “an episodic model” in which periods of “normal science” comprising periods of conceptual continuity aligned with cumulative progress, that were interrupted by periods of “revolutionary” science (the paradigm shift). The discovery of “anomalies” leads to progress in developing new paradigms. Such new paradigms pose different interpretations of old data, reject the simplicity of the previous paradigm, and initiate new research directions and novel ideas (20). His book *The Structure of Scientific Revolutions* is an extensively cited book in the social sciences. Kuhn’s ideas have been widely discussed by philosophers and historians of science (20). There is a resonance between Thomas Kuhn in 1962 (18, 19) and Nietzsche in 1886 (16), when the latter states that a paradigm becomes a dogma because its age is taken as the reason for being the truth.

Additional anecdotical references raise the problem of dogma in the sciences. An interesting observation from Samuel Hellman (21) stated that “if the hypothesis is associated with added benefits to its proponents, then a conditional premise may become a paradigm, or occasionally a dogma. Researchers who have the temerity to question this premise, may be considered heretics…” Another example is an informative editorial concerning the decision of rejection rendered by a reputable scientific journal. The author discussed the fact that the rejection of an article was not based on the article’s quality or misuse of a scientific paradigm, but rather on a dogma (22). In other words, a rejection based on a paradigm that has reached the status of a dogma, i.e., a paradogma. Paradogma in this publication is defined as a pattern or model that is so incontrovertibly true for a person or group of people that it excludes the existence and value of all other patterns or models” and further as “a world view underlying the theories and methodology of a particular scientific subject that the users see as the only world view that is of any value.”

Finally, beyond science, many studies have shown that, especially during times of uncertainty, dogmatic beliefs allay the anxiety brought on by feelings of uncertainty. A study published in the British Journal of Psychology (23) claims that “people who dogmatically do not believe in religion and those who dogmatically believe in religion are equally prone to intolerance and prejudice towards groups that violate their important values. Prejudice towards these groups may be an efficient strategy to protect the certainty that strong beliefs provide.”

Many accepted scientific hypotheses seen as realities or even dogma have finally disappeared. Besides the Copernican revolution, one of the most famous is “spontaneous generation” persisting for centuries and finally disproved by Louis Pasteur in 1862 (24). The idea that “living things can originate from nonliving materials”, i.e., spontaneous generation, has a long history, inseparably intertwined with scientific knowledge until the Pasteurian revolution in 19^th^ century and the development of microbiology as a science. Aristotle (384–322 BC), the greatest authority in Antiquity, asserted that spontaneous generation was observable in nature, and this Aristotelian influence was important in promulgating this paradigm for many centuries. Pasteur finally challenged successfully the reality of “spontaneous generation”, showing that airborne dust contained microorganisms which develop and multiply. He created a novel *in vitro* system with his famous swan-neck flask experiment in 1862. He clearly showed that there was no growth of organisms of any kind in the sterilized broth contained in his swan-neck flask; however, when he broke the neck of the flask, microorganisms were able to contaminate the broth and multiply (24). Pasteur affirmed that “life is a germ and a germ is life”. Consequently, the dogma of spontaneous generation was completely discredited by this simple but elegant experiment, following Pasteur’s critical questioning of the concept versus its scientific reality. It is notable that this experiment and subsequent observation by Pasteur triggered an enormous dispute at the Académie des Science in Paris. However, Pasteur asserted: “it must be said, belief in spontaneous generation has been a belief of all ages; universally accepted in antiquity, most discussed in modern times, and especially in our age. It is this belief that I come to fight. Its persistence through the ages worries me very little, because as you probably know the greatest errors can exist for centuries.” (25). Today the subject of spontaneous generation is simply an amusing footnote in history. This example of Pasteur is important to consider here, because it led to the discovery of septicemia. Indeed, the model of infectious diseases with microorganisms circulating in the blood may have become a model for hematogenous metastasis. This analogy is still described at present (26).

More recently, the outstanding research of Judah Folkman on tumor angiogenesis led to the hypothesis that angiogenesis was an absolute requirement for continued tumor growth, and that anti-angiogenic therapy would be established as the gold standard for controlling cancer growth and dissemination (27). Many researchers, clinicians, and pharmaceutical companies at the time believed that anti-angiogenic therapy based on the latter concepts would completely suppress tumor growth. However, this belief was short lived as this concept failed to fully explain the complex blood supply of cancer and did not lead to successful therapeutic intervention that alone could control cancer (28). In addition, the development of resistance to many antiangiogenic therapies was observed (28).

### Perspectives on the “accepted science” of tumor spread and metastases

New discoveries can facilitate the creation of a new “certain” reality. As already mentioned, the discovery of Pasteur including the presence of infectious microorganisms in the blood could be the origin of the model of circulating tumor cells producing metastases (26). Furthermore, the discovery of tumor cells within vascular lumina in autopsy cases by Billroth in concert with Pasteur’s discovery in 1863 (29) led to the long accepted paradigm (or longstanding dogma) that tumors spread through the vasculature. In 1874, by Sir William Jenner asserted: “No one disputes that cancer spreads in the course of the veins” (30). In 1889 (31) Paget's observation that: “Tumor cells (the seeds) have a specific affinity for specific organs (the soil), and metastasis does not occur by chance”, is thought to be evidence of hematogenous tumor spread, while it could be interpreted as tumor spread by another mechanism. For example, in 1858, Virchow first proposed that “Neoplasms arise in accordance with the same laws that regulate embryonic development” (32).

Gaps in knowledge are associated with different steps of the metastatic cascade. For instance, the purported processes of intra- and extravasation, the survival of cancer cells in the circulation, the hypothesis of dormancy, the embryogenic links of cancer stem cells (CSC), and, in particular, migration within the embryo, or the formation of a premetastatic niche, are concepts still poorly understood (33).

It is remarkable that since the 19^th^ century, it is not yet possible to clearly demonstrate the phenomena of intra- and extravasation. Indeed, even the most sophisticated, elegant, and very informative studies can visualize transient vascular permeability but not objective intravasation of tumor cells (34). Such observations have included only “images showing the possible fates of extravascular disseminated tumor cells in the lung parenchyma”, and finally the “fate of tumor cells could be either recirculation, apoptosis, or extravasation into the lung parenchyma” (35).

As already mentioned, the process of hematogenous metastasis is highly inefficient (2). Fidler, without contesting the hematogenous metastasis paradigm, nevertheless analyzed the gaps in knowledge of this paradigm, pointing out: “The presence of tumor cells in the circulation does not predict that metastasis will occur as most of the tumor cells that enter the blood stream are rapidly eliminated” (5). In the same paper, he reported: “The intravenous injection of radiolabeled B16 melanoma cells revealed that by 24 hours after injection into the circulation, 0.1% or less of the cells were still viable, and less than 0.01% of tumor cells within the circulation survived to produce experimental lung metastases”. Furthermore: “The presence of tumor cells or emboli distant from the primary tumor does not prove that metastasis has occurred…. the entry of tumor cells into the circulation is common and more than a million cells per gram of tumor can be shed daily”.

Notably, therapeutic (but nevertheless “experimental”) human intravenous injection of tumor cells was used *via* peritoneovenous shunts to reduce metastatic ascites in ovarian cancer (36). A very low frequency of secondary foci was observed in these patients. Although millions of tumor cells were “experimentally” directly deposited into the vena cava every 24 hours by the shunt, patients rarely developed secondary tumors and felt better. Importantly, there was no significant increase in metastasis outside of the peritoneal cavity. Thus, although there was continuous flow of millions of tumor cells into the circulation, only rare metastases to the lung (the closest capillary bed) were observed (36). Unfortunately, these observations are now seldom recognized and even more rarely discussed.

### Cancer dormancy

Dormancy has been cited in 2021 as “one of nine cancer grand challenge problems by the National Cancer Institute and Cancer Research UK (37). Cancer dormancy is a term used to explain the extensive periods of time after therapeutic treatment(s) in which patients remain asymptomatic prior to relapse. The phenomenon of dormancy assumes that metastases occur *via* “express” intravascular dissemination (37–39) (**Figure 1**). This hypothesis was suggested in 1934 by RA Willis (38), who asserted: “neoplastic cells must have lain dormant in the tissues in which they were arrested”. Dormancy, which refers to the presence in metastatic niches of dormant cancer stem cells after extravasation, is a loosely defined phenomenon that remains poorly understood (40). Despite the accumulation of data, huge “gaps in knowledge” persist: “The cellular source of late relapse in these patients is thought to be disseminated tumor cells that reactivate after a long period of dormancy. The biology of these dormant cells and their natural history over a patient’s lifetime are still largely unclear” (41). Finally, until now dormancy has not yet been demonstrated because of the lack of appropriate or convincing models (6, 7).

### Cancer stem cells (CSC) or metastasis-initiating cells

Cancer stem cells, also called metastasis-initiating cells (6), are mainly studied for their capacity to self-renew and to initiate metastasis after dormancy by growing in distant organs (6, 42). However, they generally have not been studied as to their embryonic-like capacities to migrate from the primary tumor to metastatic sites. In our view, the CSC should be also analyzed as cells with properties of embryonic stem cells migrating during organogenesis (see paragraph below).

Finally, it is important to emphasize that, despite the development of many new therapeutic agents, cell and gene therapy, radiation therapy, etc., cancer treatments have limited success and for only short periods of time once tumor cells have left the primary tumor (7, 43). The long interval to recurrence after resection and after other therapies of various types have proved very troublesome. Particularly problematic is the development of metastatic tumors that are resistant to therapy (7).

**Figure 2:**
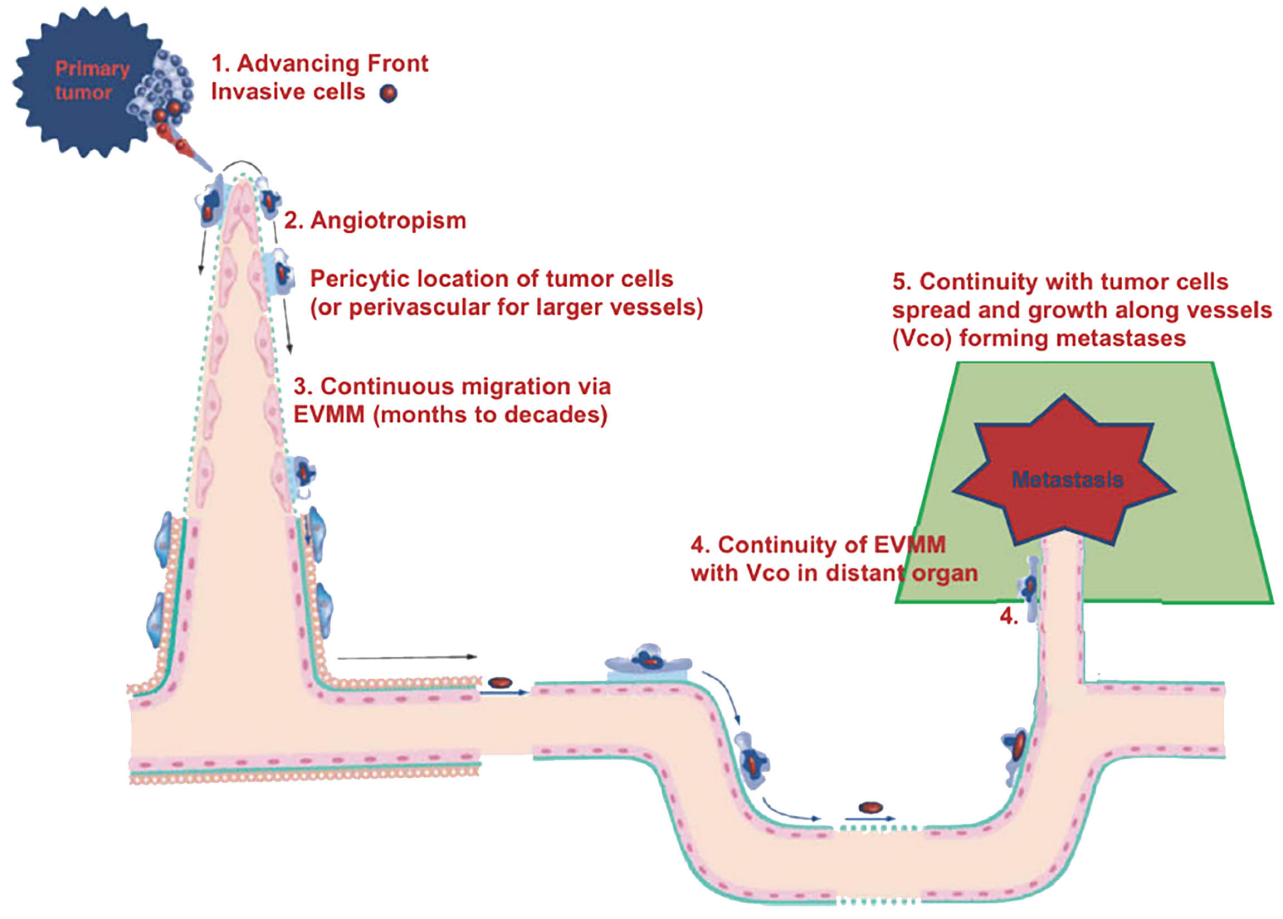
Diagram of EVMM.

We would like to offer another explanation for tumor spread and dormancy. It is possible that the dormancy period corresponds to cancer cells migrating progressively and very slowly towards metastatic sites (44), in particular along vessels, which is termed angiotropic extravascular migratory metastasis or EVMM (**Figure 2**). This is in contrast to hematogenous tumor spread and dormancy (**Figure 1**) and is discussed in more detail below.

## Angiotropic extravascular migratory metastasis (EVMM) and the continuum of vascular co-option (VCo) and angiotropic EVMM

**Figure 3:**
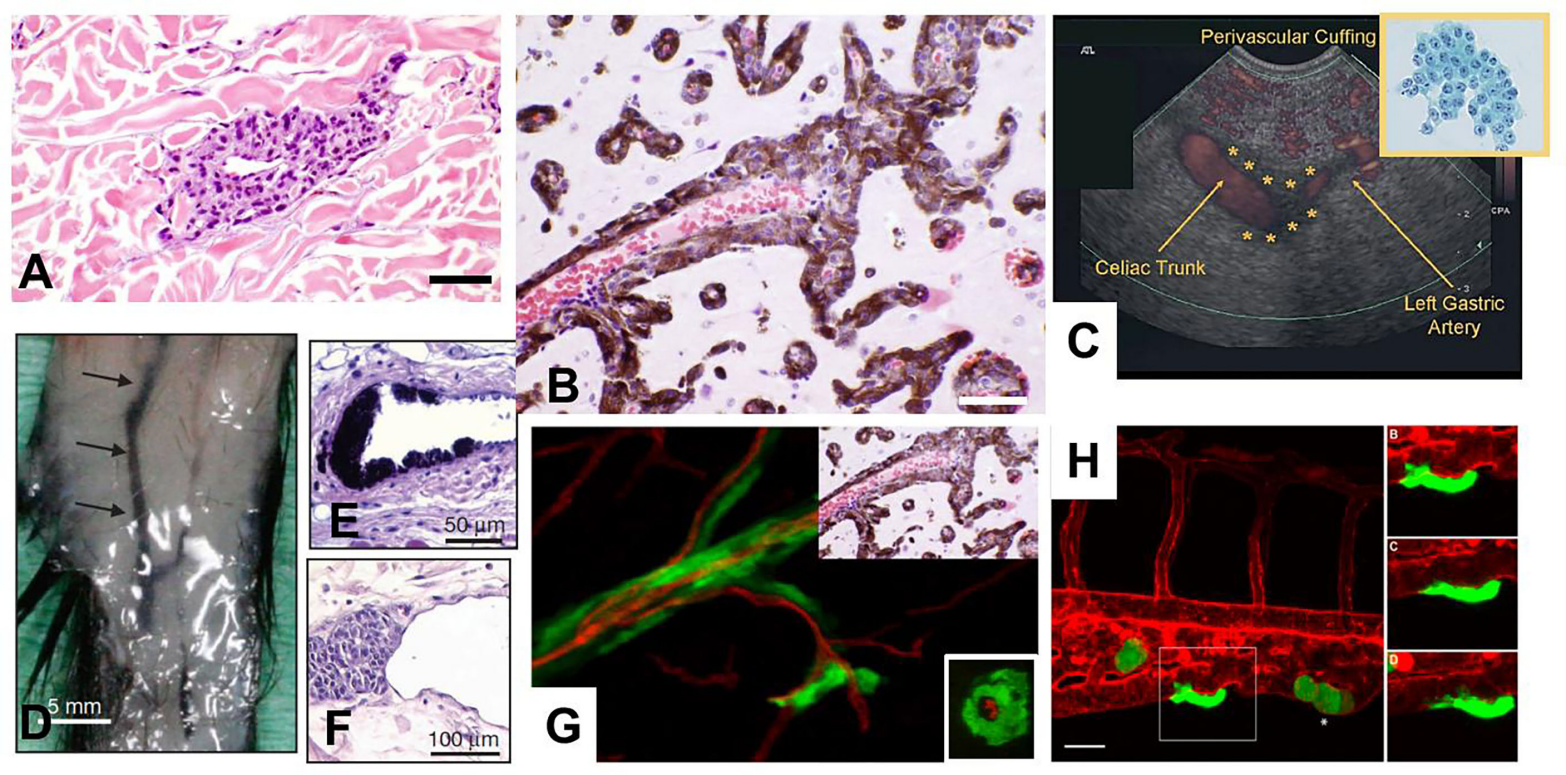
Angiotropism and angiotropic EVMM. **(A)** Human sample of melanoma showing angiotropism of tumor cells about the abluminal surface of a microvessel, some distance from the primary melanoma (about 1 mm) constituting an angiotropic microscopic satellite in the nearby dermis. Melanoma cells (dark purple cells) are cuffing the abluminal surface of the microvascular channel. Scale bars: 40 µm. **(B)** Human sample of a melanoma metastsis to the brain. Microvessels (filled by red blood cells) are extensively coated by a layer of melanin–containing melanoma cells, without intravasation, demonstrating significant angiotropism and PM. Scale bars: 100 µm. **(C)** Detection in a patient of EVMM from a remotely located pancreatic cancer in a patient (47). Linear endoscopic ultrasound and power doppler demonstrate a thin hypoechoic band (cuffing) surrounding the celiac trunk, with extension along the left gastric artery. Up-right insert: Cytologic analysis of the endoscopic ultrasound fine-needle aspiration of malignant perivascular cuffing around the celiac trunk specimen reveals a metastasis from a well-differentiated pancreatic adenocarcinoma (Papanicolaou stain). **(D–F)**. Pericytic mimicry (PC) and angiotropism in a genetically engineered mouse model. **(D)** Macroscopically visible melanoma cell expansion along 25 mm of a dermal blood vessels (arrows). Scale bars: 5 mm. **(E, F)**. Histologic analysis of angiotropism in this murine model (E, Scale bars: 50 µm), and in a human primary melanoma (F, Scale bars: 100 µm). Note the similar angiotropic images in **(E–G)**. Human melanoma cells in a murine melanoma brain model. Montage juxtaposing PM in the murine brain melanoma model (main picture) and the human sample of a melanoma metastatic to the brain detailed in B (up-right part of the image). On the main picture (murine model at 4 weeks) PM of green GFP-labeled melanoma cells are visible along red vessels (red tomato lectin. Note the correspondence of the two juxtaposed images. Insert: XZ cross-section of the murine brain vessel confirming that melanoma cells are external to the vessels, without intravasation. **(H)** Intravital observation of PM of GFP melanoma cells in a zebrafish xenograft. Angiotropism/PM of green GFP tumor cells along the external surface of the caudal vein (red tomato lectin). Scale bar 20 μm. On the right: time-lapse images of the angiotropic cell in white square taken at 0, 4 and 8 hours after the beginning of the imaging.

Some questions can be raised. For example, does the present conception of cancer progression (hematogenous spread and dormancy) show any mechanisms corresponding to those occurring during human embryogenesis? The answer seems to be no. Are spontaneous intra- or extravasation, as well as dormancy, rare events, and possibly not the “natural” events responsible of cancer progression? At the very least, can we consider alternative mechanisms of metastasis? We will briefly describe the alternative and now accepted mechanism of EVMM which has been studied for more than 25 years (45), and its potential continuum with vascular co-option (VCo) (46) (**Figure 2**). This alternative mechanism to intravascular metastatic dissemination and dormancy has been reviewed in detail in two recent publications (45, 46). Briefly, these two fields of cancer research, angiotropic EVMM (**Figure 3**) and VCo, involve tumor cells localized to the abluminal vascular surface or angiotropism (**Figure 3A**). Angiotropic EVMM constitutes a non-hematogenous mode of tumor migration and metastasis, while vessel co-option (VCo) involves a non-angiogenic mode of tumor growth. Indeed, the phenomenon of angiotropic EVMM has questioned the concept that tumor cells metastasize exclusively *via* circulation within vascular channels (48). Angiotropic EVMM involves “pericytic mimicry” which is characterized by tumor cells continuously migrating in the place of pericytes distantly along abluminal vascular surfaces toward secondary sites (45) (**Figures 2**, **3**). VCo involves tumor growth by spreading along the outside of vessels and thus challenges the concept that tumor cells grow only *via* angiogenesis (49). EVMM following VCo represents a new paradigm of cancer spread and metastatic growth, whose mechanism has strong similarities with comparable embryonic processes. Indeed, cells employ embryonic mechanisms for adhering to the outer surfaces of blood vessels (angiotropism) and spread *via* migration along the vessel outer surface (45) (**Figure 2**). This is an entirely extravascular process and does not involve entry (intravasation) into the vessel lumen. Pericytic mimicry/EVMM has primarily been studied in melanoma but seems also to be an important process in other cancers (45 and **Figure 3C**). The exact mechanism of how tumor cells migrate along the outer surfaces of blood vessels is not known but likely involves soluble as well and extracellular matrix factors (see below).

There is considerable evidence for the extravascular migration of tumor cells (45, 50). First of all, careful *in vivo* analyses of patient samples at the advancing front of primary and metastatic tumors have shown angiotropism of tumor cells and PM along the external surfaces of vessels, but not within the vessels. These observations constitute independent prognostic evidence predicting increased risk for metastasis and diminished survival. Various experimental studies, from *in vitro* coculture using tumor cells and preformed capillary-like structures to intravital imaging of PM in murine and zebrafish models, have shown that tumor cells spread *via* PM and that angiotropism is a microscopic marker of PM (45). Molecular studies have shown the role of several molecules (45), in particular laminin and more recently L1CAM (50), which are involved in cell motility and plasticity, as well as in embryonic development (50–52). In addition, pancreatic carcinoma tumor cells have been detected along the outer surfaces of abdominal veins and arteries using endoscopic ultrasound fine-needle aspiration (47, 53) (**Figure 3C**). Pancreatic cancer cells have been aspirated along the outside of major vessels in sites remote from the primary pancreatic tumor, including the celiac, hepatic, superior mesenteric, aorta, splenic, gastroduodenal and left gastric arteries, as well as the portal vein and inferior vena cava (53).

All these studies have shown these tumor cells to be quite distant from the primary tumor and demonstrate that this migration is slow and would be consistent with a long latency period.

Indeed, PM and EVMM appear to utilize a progressive step-by-step embryogenic-derived program. Many similarities exist between embryogenesis and cancer progression, including the important roles of laminins, epithelial-mesenchymal transition (EMT), and the resurrection of embryonic pathways. Notably during the first trimester of gestation, an active circulatory system does not exist even though there is cellular migration throughout the embryo during the first three months of organogenesis. Therefore, during that period, an extensive and continuous extravascular migration of embryonic stem cells takes place (45). Taken together, these processes reinforce that embryonic migratory events may recur during metastasis.

Concerning possible mechanisms underlying EVMM, several studies from us and others have shown the potential implication of several factors. For many years, we have shown the potential involvement of laminin juxtaposed between the cancer cells and the modified endothelial basement membrane (45, 50, 54). More recently, the potential implication of L1CAM in angiotropic migration has been detected (6, 48). In addition, several genes have been detected which could have a role on EVMM (45), however the precise mechanism has not yet been definitively elucidated.

It must be recalled that EVMM, if it is most often defined as an angiotropic mechanism along vessels (angiotropic EVMM), also can comprise other extravascular pathways, sometimes for considerable distances, such as described in other extravascular migratory cells and embryogenesis. Indeed, a variety of other anatomic structures are used as a scaffolding for cancer cell migration, sometimes for considerable distances. Such scaffolding includes nerves (neurotropism or perineural invasion), the peritoneum, pleura, myofibers, adipocytes, bone cavities, brain ventricles, the choroid plexus, and the glia limitans in the brain (45, 55, 56).

Finally, the perivascular location of tumor cells intrinsically associates PM to vascular co-option (VCo). The recently published perspective of a continuum of vascular co-option (VCo) and angiotropic EVMM (46) argues that VCo and angiotropic EVMM represent complementary processes and constitute a continuum of cancer progression (from growth in the primary tumor, to migration to secondary sites where secondary tumors grow to form metastases), similar to the developmental program in the embryo.

## Conclusion

The goal of this paper has been to present the possibility that a continuum of VCo and angiotropic EVMM could be considered in addition to or as an alternative to hematogenous tumor spread and tumor dormancy. In fact, to be scientifically rigorous, neither of these concepts has ever been clearly and completely demonstrated in animal models or in patients. Hematogenous metastasis, and tumor dormancy, have been and continue to be accepted as “normal science” in cancer (18). These processes are generally accepted without questioning their bases, and tend to become dogma or paradogma (18, 19, 22). On the other hand, the continuum of VCo and angiotropic EVMM, has recently been given credence and cited as an alternative to intravascular cancer dissemination (6, 57–59), but is not yet an important paradigm in the field of cancerology. It may potentially belong to a period of “revolutionary science” (18). If this mechanism becomes in the future “accepted science”, it is possible it would represent a new paradigm shift, maybe itself preceding another new period of revolutionary science.

Our goal has also been to suggest that the reader remain open-minded, avoid taking certain paradigms for granted in particular regarding tumor dissemination and dormancy, and look at the evidence in a new light that might suggest alternative mechanisms.

Fully understanding the mechanisms of tumor spread and metastatic growth is important in developing new targeted therapeutics. History and philosophy of science provide useful information and contribute to progress in science. For example, it is important to analyze and question the hypotheses, paradigms, or dogmas that are discussed by scientists. History and philosophy can provide a reservoir for hypotheses that can be verified by scientists and offer the foundations to unite several scientific concepts (60). Chemists and physicists were originally philosophers. The Scientific Revolution in Europe represented the beginning of modern science, in astronomy, mathematics, physics, chemistry and biology, and modified the perception of nature.

It is hoped that historical and philosophical debates will be replaced by a “science of understanding” (60). Finally, as stated by Thomas Kuhn: “He is the explorer of nature - the man who rejects prejudice at the threshold of his laboratory, who collects and examines the bare and objective facts, and whose allegiance is to such facts and to them alone. (…). To be scientific is, among other things, to be objective and open-minded” (19).

## Data availability statement

The original contributions presented in the study are included in the article/supplementary material. Further inquiries can be directed to the corresponding author.

## Author contributions

CL, RB, and HK conceived and wrote the manuscript. NC reviewed and commented on the manuscript. All authors contributed to the article and approved the submitted version.

## Conflict of Interest

The authors declare that the research was conducted in the absence of any commercial or financial relationships that could be construed as a potential conflict of interest.

## Publisher’s note

All claims expressed in this article are solely those of the authors and do not necessarily represent those of their affiliated organizations, or those of the publisher, the editors and the reviewers. Any product that may be evaluated in this article, or claim that may be made by its manufacturer, is not guaranteed or endorsed by the publisher.
